# Probiotics combined with atorvastatin administration in the treatment of hyperlipidemia: A randomized, double-blind, placebo-controlled clinical trial

**DOI:** 10.1097/MD.0000000000037883

**Published:** 2024-05-24

**Authors:** Yingjie Tian, Guang Wu, Xingsheng Zhao, Heping Zhang, Maojia Ren, Xiaopeng Song, Hao Chang, Zelin Jing

**Affiliations:** a Department of Cardiology, Heart Center, Inner Mongolia People’s Hospital, Hohhot, People’s Republic of China; b Inner Mongolia Cardiovascular Disease Clinical Research Center, Hohhot, People’s Republic of China; c Key Laboratory of Dairy Biotechnology and Engineering, Ministry of Education, Inner Mongolia Agricultural University, Hohhot, People’s Republic of China; d Department of Neurosurgery, Hohhot First Hospital, Hohhot, People’s Republic of China.

**Keywords:** gut microbiota, hyperlipidemia, probiotic

## Abstract

**Background:**

Hyperlipidemia is a common feature of chronic diseases. The aim of this work was designed to assess the role of probiotics (*Lactobacillus casei* Zhang, *Bifidobactetium animalis* subsp. *lactis* V9, and *Lactobacillus plantarum P*-8) in the treatment of hyperlipidemia.

**Methods:**

Thirty three patients with hyperlipidemia were randomly divided into a probiotic group (n = 18) and a control group (n = 15). The probiotic group was administered probiotics (2 g once daily) and atorvastatin 20 mg (once daily), and the control group was administered a placebo (2 g once daily) and atorvastatin 20 mg (once daily). Serum and fecal samples were gathered for subsequent analyses.

**Results:**

Time had a significant effect on the total cholesterol (TC), triglycerides (TG), and low-density lipoprotein-cholesterol (LDL-C) levels in the probiotic and control groups (*P* < .05). The gut microbial abundance in the probiotic group was markedly higher than that in the control group following 3-month probiotic treatment (*P* < .05). At the phylum level, probiotics exerted no notable effects on the relative abundance of *Firmicutes, Bacteroidetes*, and *Actinobacteria* but elevated that of *Tenericutes* and reduced *Proteobacteria*. At the genus level, probiotics increased the relative abundance of *Bifidobacterium, Lactobacillus,* and *Akkermansia,* and decreased that of *Escherichia, Eggerthella,* and *Sutterella* relative to the control group in months 1, 2, and 3 (*P *< .05).

**Conclusions:**

Probiotics optimize the gut microbiota structure and decrease the amount of harmful bacteria in patients with hyperlipidemia. Probiotics can influence the composition of gut microorganisms and increase their diversity and abundance in vivo. It is recommended to use probiotics combined with atorvastatin to treat patients with hyperlipidemia.

## 1. Introduction

Hyperlipidemia, referring to the existence of excess triglycerides or lipids in the blood, is deemed a high-risk factor and pivotal marker of arteriosclerosis and cardiovascular disease. Its main features include high contents of total cholesterol (TC), low-density lipoprotein-cholesterol (LDL-C), and triglycerides (TG), and low contents of high-density lipoprotein-cholesterol (HDL-C).^[1]^ In China, the prevalence of dyslipidaemia is up to 34.0%.^[2]^ Lipid metabolism disorders can result in altered regulation of the intestinal environment, eventually leading to gut microbiota dysbiosis.^[3]^ As a complex and relatively independent microecosystem in the human body, the intestinal flora forms a balanced unity with the host and environment. Accumulating evidence points to intestinal flora affecting and improving intestinal immunity, supplementing body nutrition,^[4]^ and protecting host health.^[5]^ Animal experiments have shown that the *Firmicutes/Bacteroides* levels increased, and *Bacteroides* and *Akkermansia* levels decreased in high-fat diet-fed mice.^[6]^ In line with animal experiments, epidemiological research found that *Bacteroides* and *Faecalibacterium* were the dominant bacterial genera associated with hyperlipidemia and the predominant bacterial genera was more abundant than that in non-hyperlipidemia cases.^[7]^ Clinical studies have confirmed that dysbiosis in patients with hyperlipidemia is mainly manifested as decreased dominant bacteria, such as *Escherichia coli, Lactobacillus*, and *Bifidobacterium*, and increased nondominant bacteria, such as *Bacteroides* and *Enterococcus*.^[7,8]^ These studies revealed that gut microbiota can alter gut microbiota in the diversity and abundance and participate in host lipid metabolism.

Probiotics act as a crucial part in hyperlipidemia as they could severely reduce the lipid contents of TC, TG, and LDL-C and also ameliorate the gut microecological balance in humans and animals.^[9,10]^ Atherosclerosis is a slowly progressive inflammatory disease caused by a lipoprotein that leads to the formation of atherosclerotic plaques within the arterial tree.^[11]^ The low-density lipoprotein (LDL)-cholesterol is associated with development of atherosclerosis, endothelial dysfunction and arterial plaques that represent the potential cause of CAD and stroke.^[12]^ Atorvastatin is one of the most worldwide prescribed statins, it was observed to reduce LDL-C, which is associated with cardiovascular mortality and morbidity.^[13]^ A 12-week, randomized, double-blind, placebo-controlled investigation with a medication mix containing the probiotic *Bifidobacterium longum* BB536 and red yeast rice extract markedly downregulated the levels of TC, LDL-C, and non-HDL-C, indicating the potential of probiotics to downregulate blood lipid levels.^[14]^ Chen et al established a hyperlipidemic rat model for exploration of the role of the gut microbiota in host lipid metabolism, and found a notable elevation in *Lactobacillus* spp*., Bifidobacterium* spp*., Bacteroides* spp*.,* and *Enterococcus* spp. in the gut of a hyperlipidemic rat model after a 28-day treatment with *Lactobacillus rhamnosushsryfm* 1301 and fermented milk.^[15]^ These results show the ability of probiotics to ameliorate TC, TG, LDL-C, and HDL-C levels via facilitating gut microbiota growth and modulating relevant metabolites.

The positive benefits of compound probiotics for humans are higher than those of single probiotic strains. Alisi et al reported that VSL#3 (a combination of 8 probiotic strains), can improve obesity, diabetes symptoms, and nonalcoholic fatty liver disease through gut microbiota regulation.^[16]^ Presently, our study group is conducting a clinical experiment on the treatment of hyperlipidemia with compound probiotics. Probiotics consisting of *Lactobacillus casei* Zhang, *Bifidobactetium animalis* subsp. *lactis* V9, and *Lactobacillus plantarum P*-8^[17]^ were used for observation of altered gut microbiota in patients with hyperlipidemia and exploration of the association of blood lipid changes with the gut microbiota. Results from these experiments provide the theoretical basis and relevant evidence for the clinical application of the lipid-regulatory effect of probiotics.

## 2. Materials and methods

### 2.1. Patients

This work was a randomized, double-blind, placebo-controlled, and parallel-group trial (RCT) carried out at Inner Mongolia People’s Hospital from January 2019 to December 2019. Of the 49 patients with hyperlipidemia, 16 patients in both groups were missing follow-up, and 33 patients (aged 55.57 ± 10.04 years) were successfully recruited (probiotic group: n = 18, control group: n = 15) and completed the full study. Inclusion criteria included hyperlipidemia with TC ≥ 6.2 mmol/L, TG ≥ 2.3 mmol/L or LDL-C ≥ 4.1 mmol/L^[18]^ and no significant past medical history. The exclusion criteria included pregnancy, tumors, infectious diseases, severe anemia, serious liver and kidney dysfunction, heart failure, stroke, autoimmune diseases, acute inflammation, infections, or trauma. Informed consent signatures from all study subjects were obtained, and the protocol was sanctioned by the Internal Review Board of Inner Mongolia People’s Hospital. This research has been reviewed by the Ethics Committee of the People’s Hospital of Inner Mongolia Autonomous Region, with approval number 202202206L and approval date of June 14, 2022.

### 2.2. Clinical procedures and evaluation criteria

The patients were assigned to 2 groups based on the random digital table method: the probiotic + atorvastatin group (probiotic group) and the placebo + atorvastatin group (control group). The probiotic group was administered atorvastatin and compound probiotic which contains 3 different strains, including *L casei* Zhang, 3 × 10^6^CFU/g; *B animalis* subsp. *Lactis* V9, 4 × 10^6^CFU/g, and *L plantarum P*-8, 3 × 10^6^CFU/g, and the dosage of atorvastatin and probiotics were 20 mg and 2 g once a day, resepectively. The placebo group was administered 20 mg atorvastatin and 2 g placebo once daily. Both groups of drugs had blank packaging with the same appearance, and digital numbers were used to distinguish the different drugs. Physical examination, as well as evaluation of BP and plasma lipid levels was conducted every 1 month.

Physical indicators included height, weight, and waist circumference (WC). The patients removed their shoes and hats and stood on a calibrated scale for their height and weight measurements. WC was calculated by circling the abdomen horizontally through the midpoint of the line between the anterior superior iliac spine and 12th costal margin. The body mass index (BMI) was introduced to categorize patients into normal (BMI < 24 kg/m^2^) and overweight (BMI ≥ 24 kg/m^2^) groups.^[19]^ WC was divided into high WC (male: WC ≥ 90 cm; female: WC ≥ 80 cm), and normal WC (male: WC < 90 cm; female: WC < 80 cm).^[20]^ Waist to height ratio (WHtR) was computed as “WC (cm)/height (cm),” and it was categorized into 2 groups: high WHtR (male: WHtR ≥ 0.53; female: WHtR ≥ 0.50) and normal WHtR (male: WHtR < 0.53; female: WHtR < 0.50).^[21]^ Blood pressure (BP) measurement was conducted following a 5 minutes rest, and again 2 minutes after the completion of the first measurement; the 2 datasets were averaged.^[22]^ The diagnostic standards of hypertension were “systolic blood pressure (SBP) ≥ 140 mm Hg and/or diastolic blood pressure (DBP) ≥ 90 mm Hg (1 mm Hg = 0.133 kPa).” Fasting venous blood samples (0, 1, 2, and 3 months of treatment) were examined within 1 hour of collection to detect plasma lipids, including TC, TG, and LDL-C. Faecal samples (0, 1, 2, and 3 months of treatment) were collected in hospital in a 50 mL cryopreservation tube filled with 15 mL DNA cryopreservation solution and kept at −80°C.

### 2.3. 16S ribosomal RNA sequencing

Extraction of total DNA from fecal samples was realized via commercial DNA extraction kits (Tiangen Biotech Co., Ltd., Beijing, China) under the supplier’s directions. The 16S rRNA gene fragment was amplified through polymerase chain reaction (PCR) with the following primers (Provided by Synbio Technologies): 27F: 5′-AGAGTTTGATCMTGGCTCAG-3′; 1492R: 5′-ACCTTGTTACGACTT-3′. For PCR amplification, 5 μg of template DNA, 10.5 μg of NEW (Beijing TianGen Biotechnology Co., Ltd), 15 μg of KOD ONE MM (Beijing Biolink Biotechnology Co., Ltd), 3 μg of barcode prime pair were employed in a 30 μg reaction mixture. The following PCR cycling conditions were applied: 95°C, 2 minutes; then 98°C, 10 seconds, 25 cycles (denaturation); 55°C, 30 seconds (annealing); 72°C, 90 seconds (elongation); finally 72°C, 2 minutes (extension). Bioinformatics analysis of gut microbiota was performed as previously described.^[17]^

### 2.4. Statistical analysis

Statistical Package for Social Sciences (SPSS) software (version 22.0, IBM, Armonk, NY, USA) was employed for all statistical analyses. Probability values (*P*) of < .05 denotes statistical significance. Normally distributed data were presented as mean ± standard deviation, Student’s *t* tests were applied to assess the difference between 2 groups. If the data were non-normally distributed or nonhomogeneous, the median and 25th percentile −75th percentile (interquartile range) was presented and the Wilcoxon rank sum test, a nonparametric test, was employed. Categorical variables were exhibited as percentages or frequencies, and a Chi-square test was applied for group comparisons. Two-way repeated-measures analysis of variance (ANOVA) was applied to determine differences between 2 groups with changes related to the levels of blood lipids, TC, TG, and LDL-C. All significance tests were 2-sided.

## 3. Results

### 3.1 Baseline characteristics and the effect of intervention on plasma lipids

In total, 33 patients participated in this study, which were assigned to the probiotic group (n = 18) and control group (n = 15) at random. Baseline characteristics, including physical indicators (e.g. height, weight, BMI, and WC), BP (e.g. SBP and DBP), and the levels of plasma blood lipids (including TC, TG, and LDL-C) of both groups were balanced (*P* > .05; Table 1).

**Table 1:** Baseline characteristics of the study subjects.

Characteristic	Probiotic group (n = 18)	Control group (n = 15)	*t/χ^2^*	*P* values
Age (yr)	56.93 ± 11.94	51.50 ± 9.45	0.997	.326
Height (cm)	163.00 ± 8.75	166.00 ± 8.57	−0.334	.741
Weigh (kg)	64.20 ± 8.33	72.65 ± 15.31	−1.630	.113
BMI (kg/m^2^)	24.10 ± 1.81	26.13 ± 3.56	−1.949	.060
WC (cm)	85.33 ± 7.02	88.95 ± 9.02	−0.979	.335
WHtR	0.39 ± 0.03	0.43 ± 0.07	−1.802	.081
SBP (mm Hg)	136.93 ± 16.35	132.40 ± 16.29	0.680	.503
DBP (mm Hg)	80.86 ± 9.55	84.40 ± 10.83	−0.858	.400
TC (mmol/L)	6.25 ± 1.18	6.44 ± 0.69	−0.564	.577
TG (mmol/L)	2.27 (1.65–4.21)	2.16 (1.52–3.23)	123.00	.664
LDL-C (mmol/L)	3.78 ± 1.16	4.01 ± 0.95	−0.812	.423

PS: BMI = body mass index, DBP = diastolic blood pressure, LDL-C = low-density lipoprotein-cholesterol, SBP = systolic blood pressure, TC = total cholesterol, TG = triglycerides, WC = waist circumference, WHtR = waist to height ratio.

**Figure 1.**
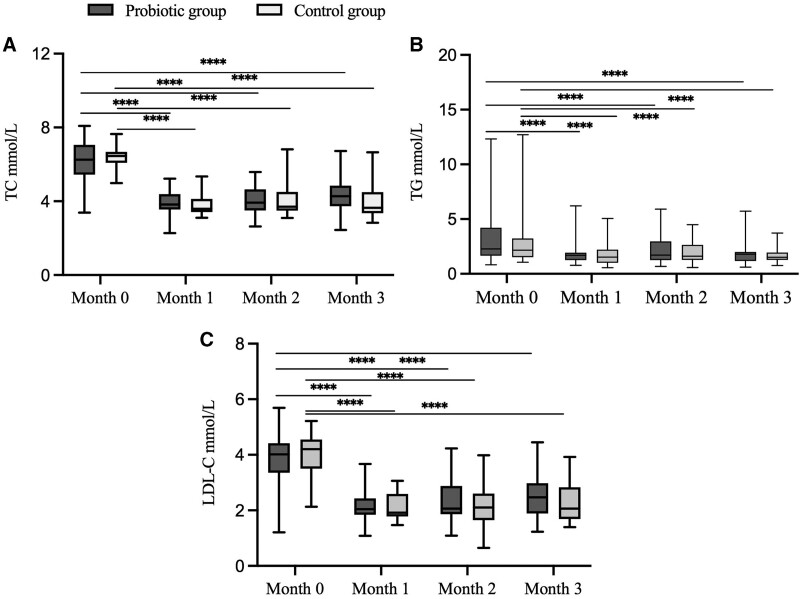
Effect of different time intervention on plasma lipid levels. Plasma lipid levels of (A) total cholesterol, (B) triglycerides, and (C) low-density lipoprotein-cholesterol is presented. Distribution of differences is shown as box-plots, with the parallel lines and whiskers representing the median and min to max values, respectively. The box-plot extends from the 25th to 75th percentile. Two-way repeated-measures ANOVA was used. ****P < .0001. ANOVA = two-way repeated-measures analysis of variance.

TC, TG, and LDL-C levels in both groups were evaluated using 2-way repeated-measures ANOVA. Time observably affected TC, TG, and LDL-C levels in the probiotic and control groups (*P* < .05). Pairwise comparisons of TC, TG, and LDL-C levels of the 2 groups exhibited notable differences between month 0 compared with months 1, 2, and 3 (Fig. 1). The effects of the group treatments and group-time interactions were not significant (*P* > .05; Table 2).

**Table 2:** Effect of different time intervention on plasma lipid.

Index	Month 0		Month 1		Month 2		Month 3		Group		Time		Group*time	
	Probiotic group	Control group	Probiotic group	Control group	Probiotic group	Control group	Probiotic group	Control group	*F*	*P*	*F*	*P*	*F*	*P*
TC	6.25 (5.44–7.05)	6.45 (6.08–6.67)	3.82 (3.55–4.38)	3.59 (3.41–4.12)	3.92 (3.50–4.64)	3.70 (3.49–4.50)	4.27 (3.73–4.85)	3.64 (3.35–4.49)	0.005	.945	93.634	˂.001	1.682	.148
TG	2.27 (1.65–4.21)	2.16 (1.52–3.23)	1.69 (1.25–1,92)	1.52 (1.01–2.22)	1.70 (1.26–2.95)	1.61 (1.28–2.64)	1.79 (1.17–1.99)	1.51 (1.27–1.94)	0.191	.665	3.888	.019	0.024	.866
LDL-C	3.78 ± 1.16	4.01 ± 0.95	2.20 ± 0.64	2.12 ± 0.47	2.27 ± 0.80	2.03 ± 0.70	2.53 ± 0.97	2.21 ± 0.67	0.049	.827	53.119	˂.001	0.156	.173

PS: LDL-C = low-density lipoprotein-cholesterol, TC = total cholesterol, TG = triglycerides.

### 3.2. The effect of alpha-diversity and structure of gut microbiota

**Figure 2.**
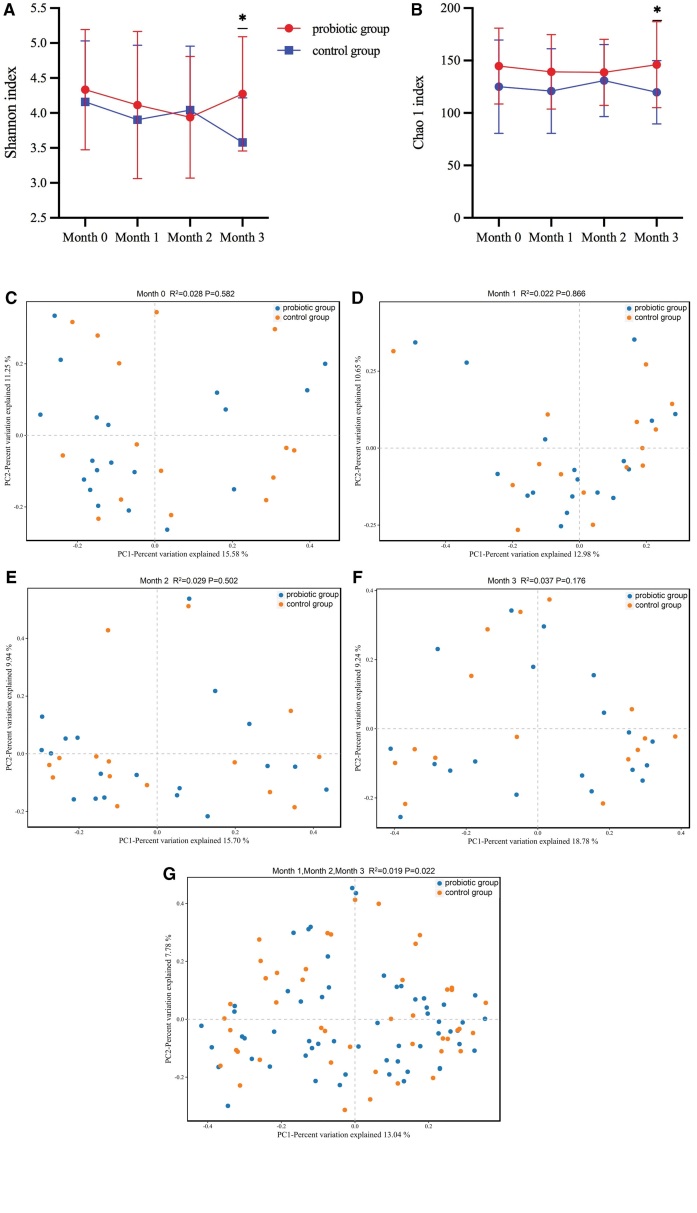
Effect of different treatments and intervention time on the alpha-diversity of fecal microbiota. Changes in the (A) Shannon index and (B) Chao1 index are presented. The Mann-Whitney test was used (*P < .05). Data points in the line charts are expressed as mean values, and error bars represent standard deviation (C, D, E, F, and G). The gut microbial community structure at each time point of the 2 groups was analyzed based on the Bray-Curtis distances using the PCoA approach. Each point represents a sample, and different groups are indicated by different colors. PCoA = principal coordinates analysis.

For alpha and beta diversity analyses, the Shannon index, Chao1 index, and principal coordinates analysis (PCoA) were applied, which can reflect the abundance, diversity, and community structure of gut microbes. Stratified analyses were performed within the 2 groups at each time point and no notable difference was observed between the 2 groups at month 0, which proved that the abundances of the 2 groups were balanced and comparable (Fig. 2A and B; *P* > .05). Cross-group comparisons of changes in gut microbiota revealed markedly elevated abundance of the probiotic group relative to that of the control group after taking probiotics for 3 months (*P *< .05), indicating that probiotic intake can enhance gut microbe diversity and abundance in vivo.

The gut microbe structure at each time point in the 2 groups was analyzed based on the Bray–Curtis distances using the PCoA approach (Fig. 2C–G). The 2 groups exhibited no observable difference at month 0, demonstrating the comparability of the gut microbe structure in both groups (Fig. 2C). Moreover, no notable distinction was detected between the groups in months 1, 2, or 3 (Fig. 2D–F). However, the overall microbial community structure was markedly changed after probiotic intervention (*P* < .05), indicating that probiotics can affect the composition of microorganisms in the gut.

### 3.3. Probiotic intervention changed gut microbiota composition

**Figure 3.**
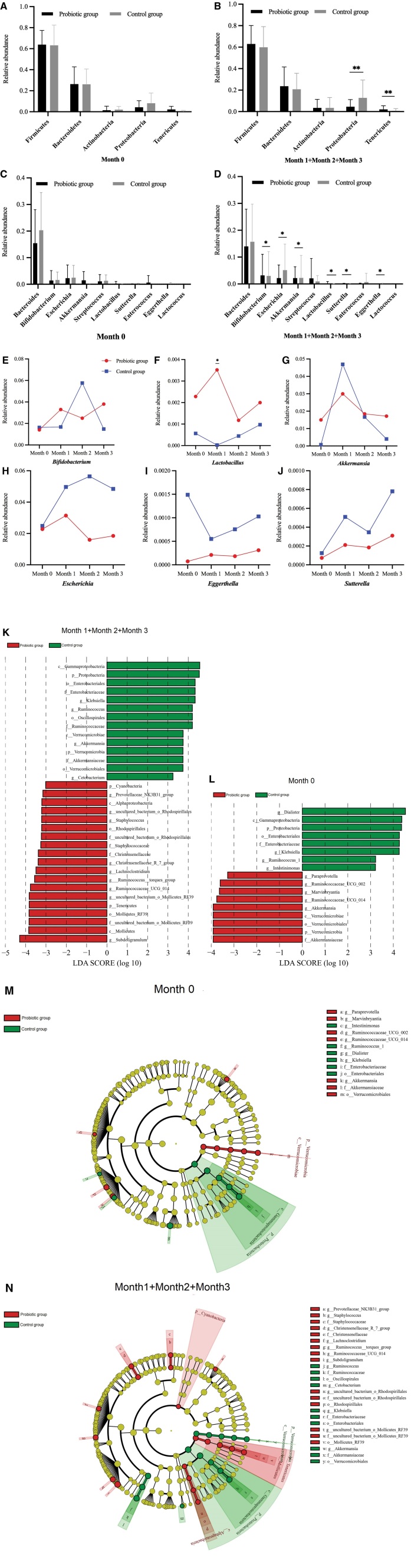
Effects of different treatments and intervention times on gut microbiota composition. Changes in the relative abundance of different phyla and genera between the 2 groups in months 0 (A), 1 (B), 2 (C), and 3 (D). Changes in the relative abundance of significant differential genera between the 2 groups at each time point are presented in E, F, G, H, I, and J. Distribution histogram based on line discriminant analysis (LDA), with a log LDA score above 3.0 (K, L). The length of the histogram represents the impact of different species which is the LDA score. Different colors represent species in different groups. Line discriminant analysis effect size of evolutionary clades. The circles radiating from the inside to the outside of the evolutionary cladogram represent the taxonomic levels from phylum to species (M, N). Each small circle at different taxonomic levels represents a classification under that level, and the diameter of the small circle is proportional to the relative abundance. Yellow represents no significant difference between species, other species with differences are colored according to the group with the highest abundance of the species. Different colors represent different groups, and nodes with different colors represent the microbial groups that play an important role in the group represented by the color. LDA = line discriminant analysis.

To further explore altered gut microbiota composition in patients suffering from hyperlipidemia, the characteristic flora was analyzed in the different groups and at the various time points. First, at the phylum and genus levels, we compared the alterations in gut microbiota composition between the 2 groups before probiotic administration. Here, we detected no notable group difference, indicating that the 2 groups were balanced and comparable (*P* > .05; Fig. 3A and C). At the phylum level, the probiotic did not change the relative abundance of *Firmicutes* (62.8% and 59.8%, respectively), *Bacteroidetes* (23.5% and 17.9%, respectively), and *Actinobacteria* (3.3% and 7.9%, respectively) but enhanced that of *Tenericutes* (1.9% and 0.6%, respectively) and reduced *Proteobacteria* (4.4% and 12.8%, respectively; Fig. 3B) relative to the control group. At the genus level, probiotic administration elevated the relative abundance of *Bifidobacterium, Lactobacillus, Akkermansia* and reduced that of E*scherichia, Eggerthella, Sutterella* at months 1, 2, and 3 (*P* < .05; Fig. 3D) relative to the control group. We compared the different bacteria at every time point to find no significant difference in bacteria except for *Lactobacillus* (Fig. 3F) and in the abundance of bacteria in each genus at each time point (Fig. 3E, G–J). Nevertheless, probiotics group showed higher abundance of *Bifidobacterium, Akkermansia,* and *Lactobacillus* than the control group (Fig. 3). In contrast, the relative abundance of *Escherichia, Eggerthella,* and *Sutterella* declined in the probiotic group relative to that in the control group at all time points. To some extent, probiotics can optimize gut microbiota composition and reduce harmful bacterium quantity in patients with hyperlipidemia.

The differentially abundant taxonomic features at the genus level were evaluated based on line discriminant analysis effect size. This revealed higher expression abundance of *Mollicutes, Tenericutes, Subdoligranulum, Staphylococcus, Rhodospitillales, Christensenellaceae,* and *Lachnoclostridium*, as well as lower *Oscillospirales, Ruminococcaceae,* and *Verrucomicrobiae,* in the probiotic group (Fig. 3I and J).

## 4. Discussion

There is evidence showing that, the gut microbiota, as an environmental factor, have important implications for hyperlipidemia.^[23]^ The potential of probiotics to preserve blood lipid levels is garnering increasing interest. For example, a 12-week treatment with *L plantarum* Q180 for effectively reduced the levels of LDL-C and apolipoprotein (Apo)B-100.^[10]^ *L curvatus* HY7601, as well as *L plantarum* KY1032 downregulated TG.^[24]^ The probiotic *B longum* strain BL1 reduced TC and TG levels.^[25]^ The current study performed double-blind RCT using patients with hyperlipidemia with the aim of observing altered intestinal flora and exploring the relationship between intestinal flora and blood lipid changes using a probiotic consisting of *L casei* Zhang, *B animalis* subsp. *lactis* V9, and *L plantarum P*-8.

The regulatory role probiotics in blood lipids was investigated by assessing the levels of TC, TG, and LDL-C. Both the control and probiotic groups showed downregulated TC, TG, and LDL-C at months 1, 2, and 3 in contrast to month 0; however, the degree of decline in the probiotic group was not notable relative to the control group. Similar to our study, it was found that serum TC decreased significantly more when consuming probiotic yoghurt compared with ordinary yoghurt, and no notable changes in other blood lipid indicators were detected.^[26]^ Previous research found unchanged TC, TG, and LDL-C levels when comparing the blood lipid levels of probiotics and placebo groups in patients with diabetes.^[27]^ However, contrary to the multiple studies,^[28,29]^ we would investigate the gut microbiota composition and diversity to explain these differences.

In the case of there being no notable changes in basal conditions, the comparison was made between the alpha-diversity and community structure of the gut microbiota in both probiotic and control groups. We found that the probiotic group had notably higher gut microbial abundance than the control group following a 3-month probiotic administration. Significant variances were observed in the gut microbial community structure between the 2 groups following probiotic treatment, indicating that probiotics can enhance gut microbe diversity and abundance in vivo.

The gut microbiota principally comprises 4 phyla: *Firmicutes, Bacteroidetes, Actinobacteria*, and *Proteobacteria*, among which, *Firmicutes* and *Bacteroidetes* act as the dominant controllers.^[30,31]^ There are conflicting findings concerning the regulation of gut microbiota abundance by probiotics. It was found that treatment with *L plantarum* LC27 and/or *Bifidobacterium longum* LC decreased the populations of *Firmicutes* and *Proteobacteria* in the high-fat diet-indcued gut microbiota, along with reduced fecal lipopolysaccharide production.^[29]^ Previous study demonstrated that *Saccharomyces boulardii* CNCM I-745-treated animals displayed markedly smaller amounts of *Firmicutes* and *Tenericutes,* and significantly larger amounts of *Proteobacteria, Lentispharerae* and other unknown phyla.^[28]^ In contrast, other research did not find changes in intestinal flora after probiotic intervention.^[32]^ Our study indicated significantly reduced relative abundance of *Proteobacteria* and elevated *Tenericutes* in the probiotic group, and the 2 groups showed no obvious difference in that of *Firmicutes, Bacteroidetes*, and *Actinobacteria*.

The majority of human probiotic microorganisms fall into the 5 genera, including *Lactobacillus, Bifidobacterium, Lactococus, Streptococcus,* and *Enterococcus*.^[33]^ Currently, the most commonly utilized probiotics consist of *Lactobacillus* and *Bifidobacterium. Lactobacillus* conduces to hydrogen peroxide production, which curbs the excessive growth of various other pathogens (e.g., group B *Streptococci, Escherichia coli*, and *S aureus*).^[34]^ *Bifidobacterium* can promote the growth of other healthy bacteria by acidifying the intestinal microenvironment and improving intestinal metabolism by accelerating lipid metabolism to treat high-fat diet-triggered obesity.^[35]^ *Akkermansia* contributes to the decrease of fat accumulation by lowering both liver and serum cholesterol levels.^[36]^ A previous study which used hypercholesterolaemic mice revealed that oral administration of *Enterococcus faecalis* ATCC19433 affected the gut microbiota composition and enhanced the amounts of intestinal *Akkermansia, Bifidobacterium*, and *Lactobacillus*.^[37]^ Our work indicated increased amount of *Lactobacillus, Bifidobacterium* and *Akkermansia* in the probiotic group relative to that in the control group. Therefore, we speculated that probiotics could elevate gut microbe abundance and regulate the balance in gut microbes. *Eggerthella* participates in the metabolism of endogenous lipids and has a positive correlation with levels of plasma cholesterol.^[38]^ A study suggested that probiotic intake might be beneficial to the improvement of blood lipids via downregulating *Eggerthella* spp. levels.^[10]^ Elevated relative abundance of some gut bacteria, such as *Bacteroides, Escherichia,* and *Sutterella*, has been detected in high-fat diet-fed rats.^[15]^ Our work found that administering the probiotic downregulated the relative abundance of *Escherichia, Eggerthella,* and *Sutterella* in contrast to the control group in months 1, 2, and 3 (*P* < .05), and the probiotic group exhibited falling *Escherichia, Eggerthella,* and *Sutterella* relative abundance relative to the control group across all time points. In addition, we found that the abundance of *Mollicutes, Tenericutes, Subdoligranulum, Staphylococcus, Rhodospitillales, Christensenellaceae,* and *Lachnoclostridium* increased in the probiotic group with *Oscillospirales, Ruminococcaceae,* and *Verrucomicrobiae* decreased, further indicating that probiotics can enrich gut microbiota diversity. According to the above results, we can estimate how long the healthy state of the gut microbiota will last once probiotics administration has stopped. This may be a goal for future research. Nevertheless, this study has certain limitations. Due to the impact of the coronavirus epidemic, the number of patients visiting the hospital has decreased significantly, which resulted in difficulties collecting samples from patients with hyperlipidemia. This has led to the sample size not achieving the expected effect.

In conclusion, probiotics have good application prospects for the treatment of hyperlipidemia. However, different probiotic strains and animal models need to be used to conduct more long-term animal experiments and large-sample, multi-center clinical trials. This will help to further standardize and confirm the efficacy of probiotics in clinical applications, and to confirm the legitimacy of probiotic lipid-lowering therapy scientifically.

## Acknowledgments

Currently Consent for publication

## Authors contribution

**Data curation:** Yingjie Tian, Zelin Jing.

**Funding acquisition:** Guang Wu, Yingjie Tian.

**Investigation:** Maojia Ren, Xiaopeng Song, Hao Chang.

**Methodology:** Yingjie Tian, Guang Wu, Zelin Jing.

**Project administration:** Heping Zhang, Xingsheng Zhao, Guang Wu.

**Resources:** Guang Wu, Xingsheng Zhao, Heping Zhang.

**Supervision:** Guang Wu, Xingsheng Zhao.

**Writing – original draft:** Yingjie Tian, Zelin Jing.

**Writing – review & editing:** Yingjie Tian, Zelin Jing.
